# Defensive Medicine Attitudes and Associated Factors Among Physicians Working in Obstetrics and Gynecology Clinics in Istanbul, Türkiye: Cross-Sectional Analytical Study

**DOI:** 10.2196/105255

**Published:** 2026-09-30

**Authors:** Cüneyt Destan Cenger, Giray Kolcu, Mehmet Ali Malkoç, Birgül Tüzün, Meral Çevik Doğan, Tuğba Saraç Sivrikoz

**Affiliations:** 1 Department of Forensic Medicine, Istanbul University, İstanbul, Turkey; 2 Department of Family Medicine, Süleyman Demirel University, Isparta, 32260 Çünür, Turkey, 90 5055306179; 3 Department of Medical Education and Informatics, Süleyman Demirel University, Isparta, Turkey; 4 Department of Forensic Medicine, Istanbul Yeni Yüzyıl University, İstanbul, Turkey; 5 Department of Obstetrics and Gynecology, Tuzla State Hospital, İstanbul, Turkey; 6 Department of Obstetrics and Gynecology, Istanbul University, İstanbul, Turkey

**Keywords:** defensive medicine, obstetrics and gynecology, malpractice, medicolegal risk, physicians, cross-sectional study

## Abstract

**Background:**

Defensive medicine (DM) is particularly relevant in obstetrics and gynecology (OB/GYN), where physicians face substantial medicolegal risks and malpractice-related pressures. Defensive practices may involve cost-increasing diagnostic or therapeutic behaviors and avoidance of high-risk patients or procedures. Identifying factors independently associated with DM may help inform organizational and medicolegal interventions.

**Objective:**

This study aimed to evaluate DM attitudes among physicians working in OB/GYN clinics in Istanbul, Türkiye, and to identify professional, organizational, and medicolegal factors independently associated with these attitudes.

**Methods:**

A cross-sectional analytical study was conducted among resident physicians, specialists, and faculty members working in OB/GYN departments in Istanbul between June 2023 and February 2024. Participants were recruited using nonprobability convenience sampling through on-site visits and an online survey. DM attitudes were assessed using the 14-item Turkish Defensive Medical Practice Attitude Scale (TDMPAS). Multivariable linear regression models with heteroscedasticity-consistent HC3 SEs were used to identify factors independently associated with the total and subscale scores. Sensitivity analyses were used to assess the potential influence of the data collection modality.

**Results:**

A total of 1069 physicians participated in the study. The mean total TDMPAS score was 48.12 (SD 10.59), with mean scores of 15.65 (SD 5.21) for cost-increasing behavior, 16.07 (SD 3.24) for defensive behavior, and 16.40 (SD 5.22) for avoidance behavior. Internal consistency was good for the total TDMPAS (Cronbach α=0.880), cost-increasing behavior (α=0.879), and avoidance behavior (α=0.879) and acceptable for defensive behavior (α=0.780). In the multivariable model, dissatisfaction with working conditions (B=6.62, 95% CI 4.19 to 9.05; *P*<.001), partial satisfaction (B=3.90, 95% CI 1.60 to 6.20; *P*<.001), and a history of administrative investigation (B=3.44, 95% CI 1.37 to 5.50; *P*=.001) were independently associated with higher total DM scores. Malpractice training (B=−2.18, 95% CI −3.54 to −0.82; *P*=.002), professor status compared with resident physician status (B=−7.53, 95% CI −12.22 to −2.83; *P*=.002), and longer experience in OB/GYN (B=−0.15 per y, 95% CI −0.29 to −0.01; *P*=.03) were associated with lower mean scores. The total-score model explained 17.6% of the variance (*R*²=0.176; adjusted *R*²=0.154). The data collection modality was not independently associated with the total TDMPAS score or any subscale score and did not materially alter the primary findings.

**Conclusions:**

DM attitudes were prominent among physicians working in OB/GYN and were independently associated with professional and organizational factors. Dissatisfaction with working conditions and previous administrative investigations were associated with stronger DM attitudes, whereas malpractice training and greater professional seniority showed inverse associations with these attitudes. These findings suggest that interventions addressing working conditions, medicolegal education, and institutional support for physicians may complement financial liability protection strategies. Prospective multicenter studies are required to clarify the causal relationships and evaluate the effectiveness of such interventions.

## Introduction

Defensive medicine (DM) refers to the practice in which doctors order diagnostic tests, treatments, and procedures to shield themselves from potential lawsuits by patients rather than focusing on diagnosing and treating the patient [1,2]. Doctors may request numerous tests, procedures, or appointments, regardless of their necessity, or they may decline to treat high-risk patients to prevent legal action [3]. DM can be categorized into 2 primary types: positive DM (PDM) and negative DM. In contrast to the positive and negative concepts found in the current research, psychometrically DM is characterized by 3 subdimensions: behaviors that increase costs, defensive actions, and avoidance strategies [4].

Even with careful attention, medical practice can occasionally lead to unfavorable and unforeseen results. When faced with such situations, doctors might adopt defensive medical strategies, such as requesting additional laboratory tests and referrals, to bolster their diagnoses and excessively use resources throughout their careers [5]. Most gynecologists and obstetricians believe that malpractice lawsuits would influence how physicians perform their duties [3]. Moreover, they believed that there was an increase in malpractice lawsuits targeting their branches, prompting them to adopt a more defensive stance [6].

DM is a multifactorial phenomenon shaped by determinants at individual, relational, organizational, and environmental levels. A recent systematic review and meta-analysis found that professional seniority, previous medicolegal litigation, and organizational setting were among the factors associated with DM, supporting a multilevel rather than exclusively individual explanation of these practices [7].

DM is a contentious topic globally, with numerous studies by doctors indicating its high prevalence rates. Research has shown that in the United States, these rates range from 79% to 93%, while in Israel, they are 60% [7]. It has been observed that the most common rate in Europe (80%) is in Italy, 98% in Japan, 99.8% in Iran, and 78.38% to 93.6% in studies conducted at different times in the same province in our country [8-11]. It has been reported that 24.2% of gynecology and obstetrics physicians practiced very good, 50% good, and 25.8% moderate DM [3]. A previous study found that 62.9% of obstetricians used at least one defensive medical practice [12]. It has been reported that 71.8% of obstetricians and gynecologists have practiced DM at least once [13].

This study aimed to evaluate DM practices in obstetrics and gynecology (OB/GYN), which are considered high-risk for medical malpractice lawsuits.

## Methods

### Study Design and Participants

This study used a cross-sectional analytical research design. The study population included faculty members, specialists, and resident physicians working in gynecology and obstetrics clinics in Istanbul.

This cross-sectional analytical study included resident physicians, specialists, and faculty members working in the OB/GYN departments in Istanbul, Türkiye. Participants were recruited using a nonprobability convenience sampling strategy through on-site visits to participating institutions and the distribution of an online survey link. Physicians actively working in the OB/GYN department during the study period and who provided informed consent were eligible. Incomplete questionnaires and duplicate submissions were also excluded.

Because recruitment was based on on-site convenience sampling and distribution of an online survey link, the total number of eligible physicians who received or had access to the invitation could not be reliably determined; therefore, a formal response rate could not be calculated.

### Data Collection

The study was conducted face-to-face between June 2023 and February 2024; however, owing to the workload of physicians, 11.6% of the surveys were conducted online and 88.4% were conducted face-to-face. The questionnaire consisted of 2 parts: the first consisted of questions about the demographic data and professional characteristics of the volunteers participating in the study.

In the second part, the Turkish Defensive Medical Practice Attitude Scale (TDMPAS), which consists of positive and negative defensive medical practice questions developed by Kolcu and Özceylan [4], was used as the data collection tool [6]. The TDMPAS consists of 14 items and 3 subdimensions [4]. A Likert scale was used to complete the questionnaires. There were no negative questions in the scale. The cost-increasing behavior subscale consists of 5 items, with possible scores ranging from 5 to 25. The defensive behavior subscale consists of 4 items, with a score range of 4 to 20. The avoidant behavior subscale also consists of 5 items, with scores ranging from 5 to 25. The total score on the scale ranged from 14 to 70. Higher scores on the scale and its subscales indicate a stronger tendency toward DM. Since there is no valid cutoff point for the scale, the scores should be interpreted dimensionally rather than categorically.

### Statistical Analysis

The data obtained were entered into SPSS software (version 22.0; IBM Corp) and statistically evaluated. Descriptive statistics and frequency distributions were calculated. Continuous variables were summarized using the mean (SD) and/or median (IQR), as appropriate. Normality was evaluated using graphical methods and the Shapiro-Wilk test. Because the total TDMPAS score was not normally distributed, unadjusted 2-group comparisons of the total score were performed using the Mann-Whitney *U* test, and comparisons involving more than 2 groups were performed using the Kruskal-Wallis test. Significant Kruskal-Wallis tests were followed by pairwise post hoc comparisons with Bonferroni adjustment. These unadjusted group comparisons were conducted for the total TDMPAS score only; subscale-specific unadjusted group comparisons were not performed. The 3 subscale outcomes were evaluated separately in the multivariable regression models and in the data collection modality sensitivity analysis.

In the confirmatory analyses, statistical significance was set at *P*<.05. Multivariable linear regression analyses were performed to identify factors independently associated with the total DM attitude score and each of its 3 subscale scores. Candidate predictors were selected a priori based on clinical relevance and the previous literature. Age, years in medical practice, and years in specialty practice were assessed for multicollinearity and were not entered simultaneously when strong correlations were observed. The categorical variables were dummy-coded. The regression assumptions were evaluated using residual plots, variance inflation factors, and influence diagnostics. Heteroscedasticity-consistent HC3 SEs were used when appropriate. The results are presented as unstandardized coefficients, SEs, standardized β coefficients, 95% CIs, and *P* values. Model fit was reported using *R*², adjusted *R*², and the *F* statistic. In the shift duration calculation, 125 physicians on call were excluded.

### Ethical Considerations

Ethics committee approval for the study was obtained from the Istanbul University, Istanbul Faculty of Medicine Clinical Research Ethics Committee (dated May 2, 2023, and numbered 1745917). This study was conducted in accordance with the Declaration of Helsinki. Informed consent and written permission to publish the data were obtained from all the individuals in the study.

## Results

A total of 1069 obstetricians and gynecologists participated in the study. Demographic, professional, institutional, and workload characteristics are summarized in Table 1. Monthly on-call duration was available for 944 participants and had a mean of 74.00 (SD 55.96) hours.

**Table 1.** Participant characteristics (N=1069).

Characteristics	Value
Age (y), mean (SD; range)	37.86 (11.58; 24-83)
Gender, n (%)	
Female	682 (63.8)
Male	387 (36.2)
Marital status, n (%)	
Married	598 (55.9)
Single	420 (39.3)
Divorced/separated	48 (4.5)
Widowed	3 (0.3)
Professional title, n (%)	
Resident physician	459 (42.9)
Specialist physician	354 (33.1)
Professor	103 (9.6)
Associate professor	77 (7.2)
Assistant professor	37 (3.5)
Subspecialty resident	30 (2.8)
Lecturer	9 (0.8)
Institution, n (%)	
Training and research hospital	572 (53.5)
Public university hospital	153 (14.3)
Private hospital	133 (12.4)
Foundation university hospital	132 (12.3)
Private obstetrics/gynecology practice	44 (4.1)
State hospital	35 (3.3)
Medical practice (y), mean (SD; range)	13.37 (11.70; 0.2‐60)
Working in obstetrics/gynecology (y), mean (SD; range)	11.66 (11.28; 0.2‐60)
Weekly working hours, n (%)	
<40 hours	77 (7.2)
40‐50 hours	390 (36.5)
51‐60 hours	225 (21.0)
>60 hours	377 (35.3)
Patient workload, mean (SD; range)	
Outpatient clinic days per month	13.74 (7.16; 0‐26)
Patients examined per outpatient clinic day	41.79 (24.20; 0‐120)
Patients under responsibility in the inpatient service	31.18 (26.06; 0‐200)
Monthly on-call duration, h	74.00 (55.96; 0‐200)

Most physicians voluntarily chose Medicine 82.6% (883/1069); OB/GYN 85.4% (913/1069). Approximately half were only partially satisfied with their working conditions, while over one-third were dissatisfied. The main sources of dissatisfaction were heavy workload, low salary, and long working hours, alongside issues such as patient violence, poor facilities, and insufficient training.

Malpractice and precaution training was received by 74.3% (341/459) of residents but only 41.5% (253/610) of specialists/academics (*P*<.001). Among those trained, 40.6% (241/594) found it adequate, while 59.4% (353/594) considered it inadequate. A large majority (946/1069, 88.5%) of all participants expressed a desire for in-service malpractice training at their institutions.

Most physicians perceived the risk of malpractice lawsuits as high. Nearly half (474/1069, 44.3%) considered it extremely high, while 27.1% (290/1069) rated it as very high and 22.6% (242/1069) rated it as high. Only a small minority (approximately 64/1069, 6.0%) believed that the risk was low.

The study found that malpractice-related administrative investigations were more common among specialists and academic physicians (237/610, 38.9%) than among residents (54/459, 11.8%), a statistically significant difference (*P*<.001).

Among physicians investigated for malpractice, approximately one-third reported decreased performance, while others experienced little or no change. None of the resident physicians investigated were found to be at fault. Among specialists and academic staff, the vast majority (221/237, 93.3%) were cleared, while only a small minority 5.9% (63/1069) were found at fault or remained under investigation.

Among the 291 investigated physicians, 96 (33.0%) reported that performance definitely decreased and 88 (30.2%) reported that it decreased; overall, 63.2% (184/291) reported decreased performance. Nearly half (362/778, 46.5%) said it would definitely decrease, whereas 32% (249/778) thought it would decrease. Only a small minority felt that it would not decrease or would definitely not decrease.

A total of 27.1% (290/1069) of the participants reported being sued for malpractice during their careers, whereas 72.9% (779/1069) had never faced litigation. Among resident physicians, the rate of malpractice lawsuits was notably low, with 91.9% (422/459) never being prosecuted. Among specialists and academic staff, 253/610 (41.5%) reported having been sued for malpractice. Among residents, 8.1% (37/459) were sued, and 91.9% (422/459) were not sued. Only a small minority of cases resulted in adverse outcomes with fines, while a few cases were reported as ongoing or concluded without any penalties.

It was determined that 95.5% (1021/1069) of participants thought that lawsuits filed with malpractice claims would have an impact on physician performance, whereas 4.5% (48/1069) thought otherwise.

It was found that 55.9% (598/1069) of participants had no malpractice concerns when choosing their branches, 28.5% (305/1069) had some concerns, and 15.5% (166/1069) had concerns.

In this study, 84.7% (905/1069) of physicians (319/459, 69.5% of residents and 586/610, 96.1% of specialists/academic staff) reported having compulsory financial liability insurance (CFLI) at the time of the survey. Additionally, 8.6% (92/1069) had previously held CFLI but were not insured at the time of data collection, and 6.7% (72/1069) had never obtained such insurance. Regarding perceptions, only 24.9% (266/1069) of participants felt comfortable practicing with insurance coverage, whereas 32.1% (343/1069) did not, and 43% (460/1069) reported feeling partially comfortable.

In this study, 91.1% (974/1069) of physicians believed that malpractice lawsuits had increased in recent years, 6.9% (74/1069) thought they had partially increased, and only 2% (21/1069) perceived no increase. The most frequently cited reasons included an increasing number of patients per physician (820/1069, 76.7%), media influence (819/1069, 76.6%), inadequate physical environments in hospitals and clinics (626/1069, 58.6%), and issues with the materials used (427/1069, 39.9%). Other contributing factors included the lack of clinical practice guidelines (384/1069, 35.9%), deficiencies in postgraduate (372/1069, 34.8%) and undergraduate (369/1069, 34.5%) medical education, insufficient informed consent (346/1069, 32.4%), and, to a lesser extent, insurance-related issues, lawyers, health policies, and patient-related factors.

In our study, the mean scores for cost-increasing behaviors were 15.65 (SD 5.21), defensive behaviors were 16.07 (SD 3.24), and avoidance behaviors were 16.40 (SD 5.22), and the total TDMPAS score was 48.12 (SD 10.59). The DM scores and subdimensions according to the institutions are shown in Table 2. In the present sample, internal consistency was good for the total TDMPAS (Cronbach α=0.880), cost-increasing behavior subscale (α=0.879), and avoidance behavior subscale (α=0.879), and acceptable for the defensive behavior subscale (α=0.780; Table 2).

**Table 2.** Defensive medicine attitude scale scores and internal consistency.

Scale/subscale	Mean (SD)	Mean/n	Minimum	Maximum	Internal consistency
Cost-increasing behavior	15.65 (5.21)	3.13	5	25	0.879
Defensive behavior	16.07 (3.24)	4.02	4	20	0.780
Avoidance behavior	16.40 (5.22)	3.28	5	25	0.879
Total score	48.12 (10.59)	—^a^	14	70	0.880

a Not applicable.

Sensitivity analyses were performed to evaluate whether the mode of data collection influenced the DM attitude scores. In unadjusted analyses, the total TDMPAS score did not differ significantly between participants who completed the survey online and those who completed it face-to-face (49.40, SD 8.99 vs 47.96, SD 10.77; Mann-Whitney *U*=62,845.5; *P*=.19). Similarly, no significant differences were observed for the cost-increasing behavior (*P*=.48) or defensive behavior (*P*=.43) subscales. The online group had higher avoidance behavior scores than the face-to-face group (17.71, SD 4.40 vs 16.23, SD 5.30; Mann-Whitney *U*=67,906.5; *P*=.004; Holm-adjusted *P*=.02), although the effect size was small (rank-biserial *r*=0.159).

To further assess the robustness of the primary findings, the data collection modality was added as an additional covariate to the multivariable HC3 robust regression models. Collection modality was not independently associated with the total TDMPAS score (B=0.20, 95% CI −1.52 to 1.93; *P*=.82), cost-increasing behavior (B=−0.50, 95% CI −1.31 to 0.31; *P*=.22), defensive behavior (B=0.00, 95% CI −0.63 to 0.64; *P*=.99), or avoidance behavior (B=0.70, 95% CI −0.17 to 1.58; *P*=.12). The inclusion of the data collection modality produced negligible changes in the model *R*² values and did not materially alter the magnitude, direction, or statistical significance of the primary regression coefficients. These findings indicate that the main study results were robust to the data collection mode.

Tables 3 and 4 present unadjusted comparisons of the total TDMPAS score only; unadjusted subscale-specific comparisons were not performed. The Shapiro-Wilk test indicated a nonnormal distribution of the total score (*P*=.001). Kruskal-Wallis analysis revealed a significant difference in total TDMPAS scores across professional titles (*χ*²_6_=74.99; *P*<.001). Pairwise comparisons showed significant differences, particularly between research assistants and professors, associate and assistant professors, specialist physicians and professors, and professors and subspecialty residents (*P*<.05). No significant differences were observed among the other title groups.

**Table 3.** Unadjusted comparisons of total defensive medicine scores.

Comparison	Chi-square (*df*)	*P* value	ε²
Comparison by title	74.99 (6)	<.001	0.07
According to the institution	36.34 (7)	<.001	0.03
According to the reasons for choosing the profession of medicine	33.39 (8)	.001	0.03

**Table 4.** Comparisons according to malpractice-related characteristics.

Malpractice-related characteristic	Participants, n	Score, mean (SD)	Minimum score	Maximum score	*P* value
Have you been sued for malpractice during your medical career?					.20
Yes	290	47.36 (11.81)	15	70	
No	779	48.40 (10.10)	14	70	
Malpractice lawsuits will have an impact on physician performance					<.001
Yes	1021	48.59 (10.30)	14	70	
No	48	38.25 (11.97)	14	65	
Have you heard of the concept of defensive medicine practices?					.96
Yes	926	48.07 (10.87)	14	70	
No	143	48.43 (8.56)	21	70	
Adequate in the content of the concept of defensive medicine practices					.50
Yes	415	47.64 (11.61)	14	70	
No	654	48.43 (9.89)	15	70	

The Kruskal-Wallis test showed a significant difference in total scores according to the institution of employment (*χ*²_7_=36.34, *P*<.001, ε²=0.03). In pairwise comparisons, significant differences were found between educational research hospitals and university hospitals (public), foundation university hospitals, and private obstetric practices (*P*<.05). No significant differences were observed among the other institutions.

The Kruskal-Wallis test showed a significant difference in total scores according to the reasons for choosing the profession of medicine (*χ*²_8_=33.39, *P*<.001, ε²=0.03). In pairwise comparisons, significant differences were found between those who chose medicine because of family suggestions and those who chose medicine because of their own will, teachers’ and friends’ suggestions, and high salaries (*P*<.05). No significant differences were observed for other factors (Table 3).

“Have you been sued for malpractice during your medical practice?” The questions were then analyzed. The average scores for those who were sued for malpractice were 47.36 and 48.40 for those who were not sued. The Shapiro-Wilk test showed that the data did not follow a normal distribution (*P*<.001). According to the Mann-Whitney *U* test results, there was no statistically significant difference between the 2 groups in terms of total scores (*P*=.20).

In the analysis based on the answers to the question, “Malpractice lawsuits will have an effect on physician performance,” the average score of those who thought that malpractice lawsuits would have an effect on physician performance was 48.59, while the average score of those who thought that they would not have an effect was 38.25. The Shapiro-Wilk test showed that the data did not fit a normal distribution (*P*<.001). The Mann-Whitney *U* test revealed a statistically significant difference between the groups (*P*<.001), indicating a significant difference between those who believed that malpractice lawsuits affected their performance and those who did not.

The analysis was based on the answers given to the question “Have you heard of the concept of DM practices before?” The average score of those who had heard of the concept of DM was 48.07, while that of those who had not was 48.43. The Shapiro-Wilk test showed that the data did not fit a normal distribution (*P*<.001). The Mann-Whitney *U* test revealed no significant difference in the total scores between the 2 groups (*P*=.96).

In the analysis made according to the answers given to the question, “Do you have sufficient knowledge about the content of the concept of DM practices?” The average score of those who stated that they did not have sufficient knowledge of the concept of DM was 48.43, while the average score of those who stated that they had sufficient knowledge was 47.64. The Shapiro-Wilk test showed that the data did not fit a normal distribution (*P*<.001). The Mann-Whitney *U* test showed no significant difference between the 2 groups in terms of total scores (*P*=.50) (Table 4).

Four multivariable linear regression models were constructed using heteroscedasticity-consistent HC3 SEs. Because age, total years in medical practice, and years working in the specialty demonstrated severe multicollinearity when entered simultaneously, the primary models retained years working in OB/GYN as a measure of professional experience. The analysis included 944 physicians with available numeric data on monthly on-call hours. The model for the total DM attitude score was statistically significant and explained 17.6% of the variance (adjusted *R*²=0.154; robust *F*_25,918_=6.174; *P*<.001; Table 5).

**Table 5.** Model fit statistics for multivariable linear regression analyses.

Variable	*R*²	Adjusted *R*²	Robust *F* (*df*=25,918)	*P* value
Total score	0.176	0.154	6.174	<.001
Cost-increasing behavior	0.199	0.177	8.795	<.001
Defensive behavior	0.046	0.020	1.611	.03
Avoidance behavior	0.180	0.158	7.936	<.001

Dissatisfaction with working conditions (B=6.61, 95% CI 4.19‐9.04; β=0.31; *P*<.001), partial satisfaction (B=3.90, 95% CI 1.60‐6.20; β=0.19; *P*<.001), and a history of administrative investigation (B=3.44, 95% CI 1.37‐5.50; β=0.15; *P*=.001) were independently associated with higher total scores. Malpractice training (B=−2.18, 95% CI −3.54 to −0.82; β=−0.10; *P*=.002), being a professor rather than a resident physician (B=−7.53, 95% CI −12.22 to −2.83; β=−0.21; *P*=.002), and longer experience in OB/GYN (B=−0.15 per y, 95% CI −0.29 to −0.01; β=−0.16; *P*=.03) were associated with lower total DM attitude scores. Gender, daily outpatient volume, monthly on-call hours, malpractice lawsuit history, and liability insurance status were not independently associated with the total score. Table 6 summarizes the statistically significant predictors of the total TDMPAS score; complete results for all covariates.

**Table 6.** Statistically significant predictors of the total Turkish Defensive Medical Practice Attitude Scale score.

Variable	B (95% CI)	HC3 SE	*P* value
Not satisfied with the working conditions	6.615 (4.189 to 9.040)	1.237	<.001
Partially satisfied with working conditions	3.898 (1.600 to 6.196)	1.172	<.001
Administrative investigation: yes	3.439 (1.375 to 5.504)	1.053	.001
Malpractice training: yes	−2.178 (−3.536 to −0.820)	0.693	.002
Professor (vs resident physician)	−7.526 (−12.223 to −2.830)	2.396	.002
Years working in obstetrics/gynecology (per year)	−0.153 (−0.292 to −0.014)	0.071	.03

## Discussion

### Principal Findings

In this large cross-sectional study of 1069 OB/GYN physicians, DM attitudes were common, with a mean total score of 48.12 (SD 10.59). Multivariable analysis showed that dissatisfaction with working conditions and a history of administrative investigation were independently associated with higher DM scores, whereas malpractice training, professor status, and longer specialty experience were associated with lower scores. In contrast, malpractice lawsuit history, sex, patient volume, on-call workload, and liability insurance status were not associated with the total score.

This pattern is consistent with recent evidence that DM reflects multilevel determinants spanning individual, relational, organizational, and environmental domains [7].

The explanatory power of the models also differed substantially across TDMPAS dimensions. The cost-increasing behavior and avoidance behavior models explained 17.7% and 15.8% of adjusted variance, respectively, whereas the defensive behavior model explained only 2%, despite reaching overall statistical significance (*P*=.03). This low explained variance suggests that the demographic, workload, professional, and organizational characteristics included in our models are less informative for generalized defensive behavior than for cost-increasing or avoidance-oriented responses. The defensive behavior subscale also had a relatively high mean score in relation to its possible range (16.07/20), which may limit between-participant variability. Other unmeasured factors, such as individual risk perception, previous adverse-event experiences, local institutional culture, patient expectations, specialty-specific clinical scenarios, and perceived litigation climate, may therefore account for a larger proportion of variation in this dimension.

Malpractice-related pressure should also be considered a psychological and occupational phenomenon rather than solely a legal or financial concern. Physicians involved in adverse clinical outcomes, malpractice allegations, or administrative investigations may experience anxiety, guilt, loss of professional confidence, sleep disturbances, fear of recurrent litigation, and changes in clinical decision-making. These responses have been described within the framework of medical malpractice stress syndrome and the second-victim phenomenon. Recent evidence from Türkiye has shown that medical malpractice stress syndrome and DM are closely related among obstetricians and gynecologists, highlighting the particular vulnerability of this high-risk specialty to malpractice-related psychological stress [14]. Similarly, recent research among obstetricians and gynecologists has demonstrated that surgical complications may negatively affect physicians’ mental well-being, sleep, professional confidence, and interpersonal functioning, supporting the relevance of the second-victim framework in this field [15]. Such psychological responses may contribute to both assurance-oriented defensive behaviors, including additional diagnostic testing or referrals, and avoidance-oriented behaviors, including reluctance to undertake high-risk procedures or manage high-risk patients. In the present study, the independent association between previous administrative investigations and higher DM scores may reflect not only medicolegal exposure but also the psychological and professional burdens associated with being formally investigated. These findings support the importance of institutional approaches that combine medicolegal education with peer support, structured debriefing, and psychological support for physicians involved in adverse events or malpractice-related processes.

In studies on DM among gynecologists and obstetricians, 38.7% to 90% of physicians were women, while this rate was 63.8% (682/1069) in our study [6,16]. In our study, the average working time of physicians in the specialty of OB/GYN was 11.66 (SD 11.28) years, while in another study, 14.5% of physicians worked for less than 5 years, 41.9% worked for 5 to 10 years, 43.6% worked for 10 years or more, 59.6% worked for 1 to 3 years, 21.1% worked for 4 to 9 years, and 19.3% worked for 10 years or more. In another study, the average working time in the specialty of OB/GYN was 8.3 (SD 3.1) years [6,12].

In our study, 42.9% (459/1069) of obstetricians and gynecologists were research assistants, whereas in similar studies, this rate was found to be 25% to 34.2%, and it was determined that the rate of research assistants in our study was higher than that reported in previous studies [7,12].

In our study, the average number of patients seen in outpatient clinics by OB/GYN physicians was 41.8 (SD 24.2), and the maximum number of patients seen was 120, while in another study, it was reported that OB/GYN physicians saw 50.25 (SD 9.5) patients per day in outpatient clinics, but the required number was 29.3 (SD 3.4). A research investigation across various departments revealed that physicians, on average, provided care to 47.28 patients daily [6]. Although the average number of patients seen in the outpatient clinic per day in our study was considered to be low compared to the literature, it was determined that the average number of patients seen in the outpatient clinic in our study was 42 patients per day, and the time allocated per patient in our study was 11 minutes at best. However, in clinics where 120 patients were seen in the outpatient clinic per day, the time allocated per patient was 4 minutes at best, which is insufficient.

According to a study in which 19,200 physicians from 26 different specialties participated in the United States, approximately 60% of physicians spend 13 to 20 minutes on outpatient diagnosis and treatment applications. Approximately 22% to 27% spend less than 13 minutes, and 5% spend less than 10 minutes. Approximately 23% of specialist physicians spend more than 20 minutes [17]. In our study, physicians examined an average of approximately 42 patients per outpatient clinic day, corresponding to a limited amount of time available for each patient. A review of national and international evidence concluded that outpatient visits should generally not be shorter than 10 minutes and that longer consultation times may be required for patients with complex medical conditions [18].

Medicolegal and financial liability concerns may also contribute to defensive medical practices. Çınarlı et al [19] examined defensive medical practices among health care professionals working in public institutions in Türkiye in the context of concerns about recourse compensation claims following medical errors.

The World Medical Association and the Turkish Medical Association state that the time allocated for each patient should be at least 20 minutes to provide quality health care. However, in our country, this time is below the European Union standards because of many problems in the provision of health services from beginning to end (personnel shortages, infrastructure problems, operational problems, etc). When the time allocated to patients coming without an appointment and for control is added, the time allocated per patient is reduced to 4 to 5 minutes. In fact, the recommendations of the Turkish Medical Association were not considered, and patient density was attempted to be solved by assigning 2 patients to physicians in 10 minutes through the central physician appointment system. Malpractice lawsuits for lack of diagnosis, treatment, and care have started to increase rapidly as the allocated time has decreased to 3 to 5 minutes [20].

Studies from Türkiye have reported high frequencies of defensive medical practices: among gynecologists, 64.6% (40/62) reported avoiding patients perceived as likely to litigate, while 93.5% (58/62) believed that malpractice lawsuits could affect their professional performance [3]; in Konya, 75.7% (131/173) of physicians reported increased use of imaging tests to protect themselves against malpractice allegations [21]; among resident physicians, positive and negative DM practices were reported by 98% (196/200) and 92% (184/200), respectively [22]; among physicians working in surgical disciplines, 94.2% (179/190) reported engaging in at least one positive or negative DM practice [23]; and among emergency medicine residents, 94.7% (145/153) believed that the number of malpractice lawsuits had increased, 83.7% (128/153) reported avoiding patients with a high likelihood of litigation or complex medical problems, and 100% (153/153) reported requesting additional laboratory or radiological tests to protect themselves against malpractice claims [24].

In a study conducted with OB/GYN physicians, it was reported that 8.1% to 51.4% of physicians were sued for malpractice, while this rate was 27.1% in our study, which is consistent with the literature [3,12].

It is estimated that 74.1% of OB/GYN specialists will face malpractice lawsuits by the age of 45 years and 97.2% by the age of 65 years [25]. Zhu et al [12] found that 60.8% of OB/GYN specialists had at least one medical dispute, 19% were sued, and 60.1% of those sued lost the case. In our study, 27.1% (290/1069) of OB/GYN physicians were sued, and 5.5% (16/290) of those who were sued received unfavorable results or were fined. In previous studies, 89.7% and 100% of OB/GYN physicians thought that malpractice lawsuits had increased, whereas in our study, 91.1% (974/1069) of physicians thought that malpractice lawsuits had increased in recent years, and 6.9% (74/1069) thought that they had partially increased. Our results are consistent with those of previous studies.

In a Turkish study carried out in Turkey involving 108 gynecologists and obstetricians, every participant reported an increase in malpractice lawsuits targeting their field, leading them to adopt more defensive practices [26]. A significant proportion of the participants (n=75, 69.4%) believed that informed consent forms would not provide protection in cases of medical errors. This perspective was shared by 75 individuals who doubted that such forms would shield them from potential medical malpractice claims [6]. In our study, 95.5% (1021/1069) of participating gynecologists and obstetricians expressed that malpractice lawsuits would hinder their medical practice.

In a study investigating DM among physicians in the United Arab Emirates, 54.6% were aware of PDM, 50.7% were afraid of patients and attendants, and 31.1% reported using a DM approach [23]. In 2023, another study of 1797 physicians in Egypt reported that a high proportion of participants had DM. Specifically, 89.6% reported avoiding high-risk procedures, 87.8% avoided treating high-risk patients, and 86.8% reported making unnecessary referrals to other specialists. Among the study participants, obstetricians and surgeons were most likely to have DM [27].

A study conducted among resident physicians in Turkey found that the frequencies of positive and negative DM practices were 98% and 92%, respectively, and that DM scores were significantly higher in those working in surgical sciences, those who had 8 or more shifts per month, and those who had medical malpractice lawsuits filed against them [22]. In a study of OB/GYN specialists conducted by Bourne et al [28], 13% (400/3073) of all OB/GYN specialists, 12% (239/2069) of women, and 16% (152/963) of men showed DM behavior. Regarding OB/GYN, DM was recorded in Sudan with 71.8% and in China with 62.9% [12,13].

Previous studies have reported high frequencies of DM practices among obstetricians and gynecologists in Türkiye, including avoidance of high-risk procedures and concerns about increasing medicolegal pressure [6]. Because the TDMPAS has no validated cutoff value, the present study does not estimate the prevalence of DM. Accordingly, our findings are interpreted using continuous total and subscale TDMPAS scores.

In a particular study, 71.8% (84/117) of gyn-obstetricians engaged in defensive medical practices, 41% (48/117) employed positive defensive strategies, and 30.8% (36/117) used negative strategies. Additionally, 23.1% (27/117) of the practitioners made unnecessary patient referrals, 20.5% (24/117) steered clear of high-risk procedures, 12% (14/117) ordered unnecessary tests, and 6% (7/117) prescribed unnecessary drugs. The most commonly observed actions included physicians declining to treat high-risk patients and 5.1% (6/117) of them performing off-label surgeries, such as cesarean sections, to prevent legal actions and complaints [13].

A study conducted in Iran in 2017 revealed that the occurrence of both positive and negative DM behaviors was 100% (160/160) and 80% (128/160), respectively [29]. The most frequent PDM behavior involved ordering unnecessary biopsies, imaging studies, and laboratory tests. In contrast, the most prevalent negative DM behaviors included avoiding the care of high-risk patients and declining to accept them based on their social status. The primary reasons for engaging in DM were that physicians felt unsupported and feared being complained against [29].

In 94.2% (179/190) of the cases, practitioners employed at least one form of DM, whether positive or negative [30].

In a study conducted in Turkey, it was shown that assistant physicians ordered more tests than specialist physicians did [31].

In a particular study, obstetricians and gynecologists had an average score of 48.22 (SD 10.24) on a questionnaire about DM practices, with scores ranging from 29 to 66. The attending physicians recorded an average score of 44.16 (SD 10.31), with a minimum of 29 and a maximum of 60, whereas the resident physicians had an average score of 52.29 (SD 8.52), with scores ranging from 34 to 66 [3]. In a study conducted by Kolcu and Özceylan [4] with family physicians, the DM score was found to be 40.55 (SD 8.30). In our research, the average scores from the attitude questionnaire concerning DM practices were 48.12 (SD 10.59), aligning with existing literature [3,4,6].

Our research revealed that individuals who faced malpractice lawsuits had an average score of 47.36 (SD 11.81), whereas those who did not have a mean score of 48.40 (SD 10.10). Similarly, another study found that physicians with a history of malpractice litigation exhibited lower DM scores [30]. Physicians who had faced malpractice lawsuits exhibited significantly lower DM scores, with a statistical significance level of *P*<.05 [27].

Our research revealed notable differences between research assistants and professors, associate professors and assistant professors, specialists and professors, and professors and associate professors and subdivision assistants (*P*<.05). Although no significant differences were observed among the other titles, Çalıkoğlu identified a significant variation in DTU scores across academic titles [30].

Three primary factors affected the DM scores: academic position, field of medical practice, and prior involvement in malpractice cases. Other characteristics, such as sex, age, total professional time, and length of experience in a particular area of expertise, showed no effect on DM scores [27].

In studies conducted in our country, 82.3% to 84.2% of CHD physicians stated that they had heard of the concept of DM practices before, while this rate was 86.6% (926/1069) in our study, which is consistent with the literature [3].

In previous studies, 29% to 42.7% of physicians reported that they had sufficient knowledge about DM practices, while this rate was 38.8% (415/1069) in our study, which is consistent with the literature [3,12].

Medical malpractice insurance is compulsory for all physicians in Turkey. OB/GYNs pay the highest fees for malpractice insurance premiums and are considered the highest-risk group.

Hiyama et al [10] reported that 96% of the physicians surveyed in their study had insurance to protect themselves from malpractice lawsuits. In a 2009 study conducted among physicians working in Tokat provincial center, it was reported that only 1.7% of physicians had insurance. A 2020 study conducted among emergency medicine residents reported that 87.6% of physicians had insurance at the time of the study [24]. Our research revealed that 69.5% (319/459) of resident physicians and 96.1% (586/610) of specialist and academic staff physicians, 84.7% (905/1069) overall, had CFLI at the time of the survey and maintained it continuously, which is consistent with the existing literature. Furthermore, assistant doctors have lower CFLI than specialists and academic staff doctors because they perform their daily medical duties under the guidance of specialists and academic staff. They do not face the same level of responsibility pressure as specialists and academic staff, which may lead them to believe that insurance is unnecessary during their assistance. In a study conducted by Kumtepe et al [24], 29.4% of participants believed that CFLI would facilitate their medical practice, whereas 46.4% felt that CFLI would not make their medical practice easier.

Furthermore, the reason assistant doctors have less CFLI coverage than specialist and academic staff doctors is attributed to the fact that assistant doctors carry out their daily medical duties under the guidance of specialist and academic staff doctors. Consequently, they do not face the same level of responsibility pressure as their senior counterparts, which may lead them to believe that insurance is unnecessary [11]. In our study, 24.9% (266/1069) of participants who had previously and consistently maintained CFLI felt at ease, 32.1% (343/1069) did not feel at ease, and 43% (460/1069) felt somewhat at ease while practicing their profession, which aligns with the existing literature. The low sense of security provided by CFLI, observed in our study and other studies, is attributed to CFLI’s lack of protection under criminal law.

According to one study involving 108 participants, 102 (94.4%) respondents believed that the increased involvement of lawyers in medicolegal matters contributed to the rise in litigation [26]. The high potential for financial compensation was also identified as a factor contributing to increased litigation. Furthermore, 78.7% (85/108) respondents reported inadequate equipment, in terms of both quantity and quality, at their institutions, which could potentially contribute to medicolegal problems. In addition, 38.9% (42/108) participants reported an insufficient number of support staff, including midwives and nurses, and 50% (54/108) indicated that they occasionally had to perform procedures with an anesthesia technician rather than an anesthesiologist. In our study, 820/1069 (76.7%) obstetricians and gynecologists identified an increased patient-to-physician ratio, 819/1069 (76.6%) identified the role of media coverage, and 626/1069 (58.6%) identified the physical conditions of hospitals and clinics as factors contributing to the increase in malpractice lawsuits.

The fear of being sued for malpractice has also affected physicians’ specialty preferences over the years. The specialty of gynecology and obstetrics, which was previously preferred, with high scores in national exams in our country, has fallen to the background in the ranking of physicians’ preferences in recent years, and some quotas have become research assistant positions that cannot be filled by those who are not preferred. Similarly, in the branches of general surgery and emergency medicine, many national exam quotas have remained vacant in recent years. In addition to the heavy workload during residency training and specialization in the field, physicians’ anxiety about being complained about medical malpractice reduces the rate of preference in the relevant departments. Studies have also shown that physicians consider these factors when choosing their specialty [32]. However, while 85.4% (913/1069) of the participants in our study stated that they chose the profession of medicine voluntarily, this rate was reported to be 54.7% to 77.5% in different studies conducted in our country. The physicians in our study were more likely to voluntarily choose the medical profession than those in the literature.

This study had several limitations. Its cross-sectional design precludes the establishment of temporal or causal relationships between the identified factors and DM attitudes. For example, although dissatisfaction with working conditions and previous administrative investigations were associated with higher DM scores, the direction of these relationships could not be determined from the present data. The study relied on self-reported attitudes and experiences and may therefore be subject to recall and social desirability biases. Reported DM attitudes may not necessarily correspond directly to physicians’ actual clinical behaviors. The participants were recruited using nonprobability convenience sampling. Consequently, physicians who agreed to participate may have differed systematically from those who did not participate, introducing potential selection and nonresponse bias. Moreover, the study was conducted exclusively among physicians working in OB/GYN departments in Istanbul; therefore, the findings may not be generalizable to physicians practicing in other regions of Türkiye, other health care systems, or other medical specialties. Data were collected using both face-to-face and online approaches, which could have influenced response patterns. Although unadjusted analyses indicated a small difference in avoidance behavior scores between the 2 collection modalities, sensitivity analyses demonstrated that the data collection modality was not independently associated with the total TDMPAS score or any of its subscales after adjustment for relevant covariates and did not materially alter the primary regression findings. However, the unequal distribution of participants between the 2 data collection modalities should be considered when interpreting these findings. The multivariable analyses involving monthly on-call duration were based on 944 participants with available numeric data, rather than the entire study sample, and complete case analysis may have introduced additional bias if the missing data were not completely random. Finally, this study assessed physicians’ self-reported medicolegal experiences and attitudes but did not incorporate independently verified malpractice claims, administrative records, patient outcomes, or direct measures of unnecessary investigations, procedures, or health care expenditures. Therefore, the clinical and economic consequences of DM cannot be objectively quantified. Future prospective multicenter studies incorporating objective medicolegal, clinical, and health economic outcomes are needed to confirm these findings and assess whether organizational or educational interventions can reduce defensive medical practices.

### Conclusion

DM attitudes were common among OB/GYN physicians in Istanbul and were independently associated with several professional and organizational characteristics. In particular, dissatisfaction with working conditions and previous administrative investigations were associated with higher DM scores, whereas malpractice training and greater professional seniority showed inverse associations with DM scores. These findings suggest that interventions aimed solely at financial liability protection may be insufficient and that organizational working conditions, medicolegal education, and physician support mechanisms should also be considered. Because of the cross-sectional and nonprobability sampling design, causal interpretations should be avoided. Multicenter prospective studies are needed to determine whether modifying these factors can reduce DM attitudes and practices.

## Abbreviations

CFLI: compulsory financial liability insurance
DM: defensive medicine
OB/GYN: obstetrics and gynecology
PDM: positive defensive medicine
TDMPAS: Turkish Defensive Medical Practice Attitude Scale
